# Bioactivity-Guided Isolation and Identification of New and Immunosuppressive Monoterpenoid Indole Alkaloids from *Rauvolfia yunnanensis* Tsiang

**DOI:** 10.3390/molecules24244574

**Published:** 2019-12-13

**Authors:** Li-Mei Li, Shun-Dong Shi, Yang Liu, Qiang Zou

**Affiliations:** 1 School of Pharmacy, Southwest University for Nationalities, Chengdu 610041, Sichuan, China; 2 Research Center, Chengdu Medical College, Chengdu 610500, Sichuan, China; shisd@kelun.com (S.-D.S.); scunn519@gmail.com (Y.L.); qiangzou99@gmail.com (Q.Z.)

**Keywords:** *Rauvolfia yunnanensis*, monoterpenoid indole alkaloid, 11-hydroxyburnamine, rauvoyunnanine A, rauvoyunnanine B, immunosuppressive activity

## Abstract

Three new 11-hydroxyburnamine (**1**) and rauvoyunnanines A–B (**2**–**3**), and fourteen known (**4**–**17**) monoterpenoid indole alkaloids were isolated from the total alkaloids extract of *Rauvolfia yunnanensis*, which exhibited promising immunosuppressive activity on T cell proliferation in preliminary screening. Their structures were determined by analysis of high-resolution electrospray ionization mass (HRESIMS), ultraviolet (UV) and nuclear magnetic resonance (NMR) data, and by comparison with the literature. All the alkaloids were evaluated for inhibitory activity on T cell proliferation. Among them, one new compound (**1**) and reserpine (**6**) exhibited moderate immunosuppressive activity, with IC_50_ values of 5.9 μM and 5.0 μM, respectively.

## 1. Introduction

Abnormal T cell proliferation plays a very important role in the development of T-cell mediated organ transplantation rejection and autoimmune diseases, such as systemic lupus erythematosus (SLE) and rheumatoid arthritis (RA) [1,2]. It is crucial to find immunosuppressive agents on T cell proliferation for this kind of immunopathogenesis. Some clinical immunosuppressants inhibit T cell proliferation by different modes of action [3]. For example, cyclosporine and tacrolimus are calcineurin-inhibitors [4]. Sirolimus and everolimus (Target-of-Rapamycin Inhibitors) inhibit nucleotide synthesis and azathioprine [5,6], and mycophenolic acid inhibits inosine monophosphate dehydrogenase and *de novo* GTP biosynthesis [7]. However, their side effects, such as infections and toxicities, prompt scientists to look for new immunosuppressants constantly. Medicinal plants are also useful sources of immunosuppressive agents. Triptolide obtained from the Chinese herbal plant *Tripterygium wilfordii* Hook F is widely used in East Asia for the treatment of SLE, RA, etc. [8]. Sinomenine from *Sinomenium acutum* (Thunb.) Rehd. et Wils has been used for patients with autoimmune diseases as it possesses immunosuppressive activity [9].

*Rauvolfia yunnanensis* Tsiang (Apocynaceae) is a kind of shrub, mainly distributed in the south of China, such as Yunnan, Guizhou, and Guangxi Provinces. It is a medicinal plant of Dai Nationality in Yunnan Province, and its roots have been used for the treatment of hypertension, fever, sore throat, hepatitis, nephritis, and snakebite [10]. Until now, more than twenty indole alkaloids have been obtained from *R. yunnanensis* in previous studies [11,12,13]. The most famous one is reserpine, which possesses a significant antihypertension effect [14]. It was also reported that reserpine inhibited delayed hypersensitivity and contact sensitivity responses [15]. Yohimbine in combination with berberine has an immunoregulatory effect [16]. In our ongoing search for immunosuppressive compounds from medicinal plants [17], the total alkaloid extracts of whole *R. yunnanensis* plants exhibited promising immunosuppressive activity on T cell proliferation. Therefore, a comprehensive phytochemical investigation on the total alkaloids was carried out. The isolation, structural elucidation, and immunosuppressive activity of the isolated alkaloids are described herein.

## 2. Results and Discussion

### 2.1. Identification of New Compounds

**Figure 1:**
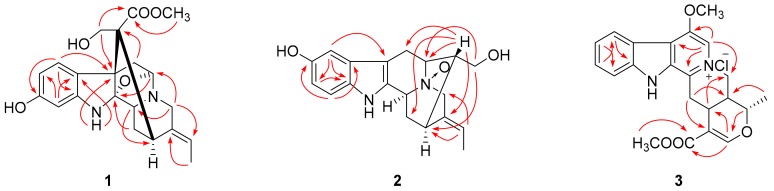
Selected HMBC correlations of compounds **1**–**3**.

**Figure 2:**
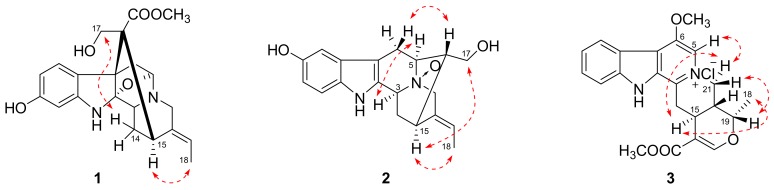
Selected NOESY correlations of compounds **1**–**3**.

Compound **1** was isolated as a yellowish, amorphous powder with [α]20D − 117.5 (MeOH, *c* 0.04). Its molecular formula was determined to be C_21_H_24_N_2_O_5_ by positive HRESIMS at *m*/*z* 385.1766 [M + H]^+^ (calcd 385.1758), corresponding to 11 degrees of unsaturation. Its UV spectrum showed absorption maxima at 207 and 293 nm, which is characteristic of a hydroindole/alkylaniline chromophore [18]. The ^1^H NMR spectrum (Table 1) exhibited an ABX spin system at *δ*_H_ 7.61 (1H, d, *J* = 8.1 Hz), 6.79 (1H, d, *J* = 1.8 Hz), and 6.71 (1H, dd, *J* = 8.1, 1.8 Hz), an ethylidene at *δ*_H_ 5.28 (1H, m) and 1.64 (3H, d, *J* = 6.5 Hz), and a methoxyl group at *δ*_H_ 3.70 (3H, s). The ^13^C NMR spectrum (Table 1) displayed 21 carbon signals including one methyl, one methoxyl, four methylenes, seven methines (four *sp*^2^ and three *sp*^3^), and eight quaternary carbons (four *sp*^2^, three *sp*^3^, and one carbonyl). The above spectroscopic evidences, along with the previously isolated alkaloid structures from this plant indicated that compound **1** was a monoterpenoid alkaloid [11]. The key HMBC correlations (Figure 1) between H-5 and C-2, H_2_-17 and C-7, H_2_-6 and C-16 suggested that compound **1** possessed a picraline-type skeleton, similar to 11-methoxyburnamine [19]. The HMBC correlations from H_2_-21 to C-19 indicated the presence of an ethylidine at C-20. In addition, the correlations of H-9 to C-7 and C-11 implied the location of a hydroxyl group at C-11. The presence of a methoxyl group at C-22 was also proved by the HMBC experiment of *δ*_H_ 3.70 to *δ*_C_ 175.6. The NOESY correlation between H_3_-18 and H-15 suggested that the configuration of C-19 should be *E*. Meanwhile, the NOESY correlations from H_2_-17 to H-14*β* indicated that the C-16 configuration is *R* (Figure 2). Finally, compound **1** was elucidated as 11-hydroxyburnamine.

**Figure 3:**
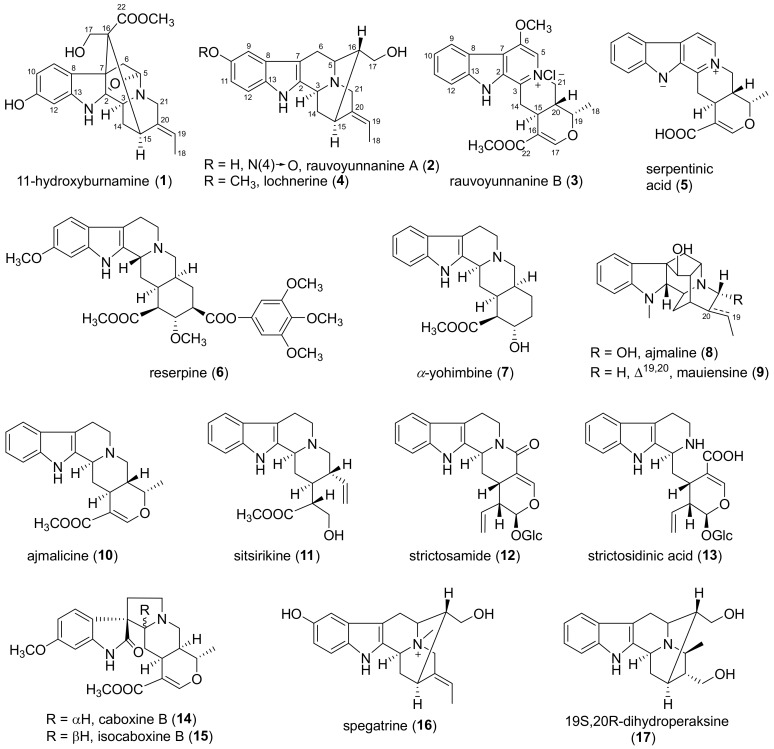
Structures of compounds **1**–**17**.

The positive HRESIMS of compound **2** showed an ion peak at *m*/*z* 327.1676 [M + H]^+^, which assigned its molecular formula as C_19_H_22_N_2_O_3_. An ABX spin system at *δ*_H_ 7.21 (1H, d, *J* = 8.5 Hz), 6.87 (1H, br s), and 6.74 (1H, d, *J* = 7.7 Hz) in the downfield of ^1^H NMR spectrum (Table 1) implied a one-substituted indole ring. Signals of an ethylidene group were present at *δ*_H_ 5.62 (1H, m) and 1.70 (3H, d, *J* = 6.5 Hz). These two substructures corresponded to ten *sp*^2^ carbon signals at *δ*_C_ 102.0–152.3 and one methyl signal at *δ*_C_ 13.2 (Table 1). On the basis of HSQC experiment, the left eight carbon signals were comprised of four methines and four methylenes. These NMR data of compound **2** were similar to those of lochnerine (**4**) [20], except that the methoxyl group in **4** was displaced by a hydroxyl group in **2**. The HMBC correlations of H-9 to C-7, C-11, and C-13, H-11 to C-9 and C-13, and H-12 to C-8 and C-10 confirmed this hydroxyl group was at C-10. Further careful comparison of the ^13^C NMR data between **2** and **4** demonstrated that the chemical shifts of C-3 (*δ* 66.8), C-5 (*δ* 70.7), and C-21 (*δ* 69.8) were remarkably downfield shifted, which indicated that **2** was an *N*-oxide. The HMBC correlations from H-16, 17 to C-5, and H-19 to C-21 confirmed this deduction (Figure 1). Although no HMBC correlations were observed for C-3, the chemical shift at *δ*_C_ 66.8 was the only left nitrogenized methine, which should belong to C-3. The relative configuration of **2** was the same as that of **4**, which was verified by a NOESY experiment (Figure 2). Thus, the structure of compound **2** was identified as shown in Figure 3 and named rauvoyunnanine A.

The characteristic UV maxima absorption bands of compound **3** were at 252 (4.38), 307 (4.07), and 367 (3.44) nm, almost identical to those of serpentinic acid (**5**). Moreover, the ^1^H and ^13^C NMR data of **3** (Table 1) were similar to those of **5** [21], but two more methoxyl groups in **3**. In the HMBC spectrum (Figure 1), one of the methoxyl group (*δ*_H_ 3.82) correlated to the carbonyl group at *δ*_C_ 168.5. The other one (3.60, *δ*_C_ 64.3) was located at C-6, which was supported by HMBC correlations from H-5 (*δ*_H_ 8.24, 1H, s) to C-3, C-6, C-7, and C-21. On the basis of HSQC, HMBC, and NOESY spectra, other substructures and the relative configuration of compound **3** were assigned as the same with compound **5**. As its molecular formula was predicted as C_22_H_23_N_2_O_4_Cl from an ion peak at *m*/*z* 437.1274 [M + Na]^+^ in HRESIMS (calcd C_22_H_23_N_2_O_4_ClNa, 437.1239), compound **3** was a chloride salt. Finally, the structure of compound **3** was determined as shown in Figure 3, and named rauvoyunnanine B.

The known compounds **4**–**17** were identified as lochnerine (**4**) [20], serpentinic acid (**5**) [21], reserpine (**6**) [13], α-yohimbine (**7**) [22], ajmaline (**8**) [22], mauiensine (**9**) [23], ajmalicine (**10**) [24], sitsirikine (**11**) [25], strictosamide (**12**) [26], strictosidinic acid (**13**) [27], caboxine B (**14**) [28], isocaboxine B (**15**) [28], spegatrine (**16**) [29], and 19(*S*),20(*R*)-dihydroperaksine (**17**) [30] by comparing their MS, ^1^H and ^13^C NMR data with those reported in the literature, respectively.

### 2.2. Immunosuppressive Activity Assay

All isolated compounds were evaluated for the inhibitory activity against human T cell proliferation according to reported protocols [17]. Compounds **1** and **6** showed immunosuppressive activity on human T cell proliferation, with IC_50_ values of 5.9 μM and 5.0 μM, respectively. Other compounds with IC_50_ > 50 μM were inactive (Table 2).

## 3. Experimental

### 3.1. General Experimental Procedures

A PerkinElmer 341 digital polarimeter was used for optical rotations. UV spectra were measured on a PerkinElmer Lambda 35 UV/VIS spectrometer (PerkinElmer, Waltham, MA, USA). NMR experiments were performed on Bruker Avance 600 MHz NMR spectrometer (Bruker Biospin Gmbh, Rheinstetten, Germany). A BioTOF-Q mass spectrometer (Bruker Daltonics, Billerica, MA, USA) was used to record HRESIMS spectra. Column chromatography (CC) was performed through glass columns packed with silica gel (200–300 mesh, Qingdao Marine Chemistry Co. Ltd., Qingdao, China), Sephadex LH-20 (40–70 μm, Amersham Pharmacia Biotech AB, Uppsala, Sweden), reversed-phase silica gel (octadecylsilyl (ODS), 50 μm, YMC Co. Ltd., Kyoto, Japan), macroporous resin D-101 (Chengdu Kelong Chemical Co. Ltd., Chengdu, China), and NH-gel (MB 100–40/75, Fuji Silysia Chemical Ltd., Kasugai, Japan). Analytical (1 mL/min) and semipreparative (3 mL/min) HPLC (Waters Corporation, Milford, MA, USA) were carried out on a Waters 2695 apparatus corresponding to a Kromasil 100–5C18 (5 μm, 250 × 4.6 mm) and a Waters XTerra RP18 (10 μm, 250 ×10 mm) columns, respectively. An Agilent 1260 apparatus coupling to an Agilent Pursuit XRs 5 C18 (5 μm, 250 × 21.2 mm) column was used for preparative (10 mL/min) HPLC (Agilent Corporation, Waldbronn, Germany). Thin layer chromatography (TLC) was performed on silica gel GF_254_ glass plates (Qingdao Marine Chemistry Co. Ltd., Qingdao, China). Spots were visualized by UV light (254 nm) and Wagner’s reagent. All solvents were analytical and HPLC grade.

### 3.2. Plant Material

The whole plants of *R. yunnanensis* were collected in October 2009, from Mengla County (21.08°–22.36° N latitude, 99.56°–101.50° E longitude, 900–1300 m.a.s.l.), XishuangBanna, Yunnan Province, China, and authenticated by Dr. Yu-Lan Peng, Chengdu Institute of Biology, Chinese Academy of Sciences. A voucher specimen (LMRY0904) was deposited at School of Pharmacy, Southwest University for Nationalities (Chengdu, China).

### 3.3. Extraction, Isolation, and Purification Procedures

The air-dried and powdered whole plants of *R. yunnanensis* (8.5 kg) were extracted as described before to yield CHCl_3_ and *n*-BuOH extractions [17]. The CHCl_3_ extraction (79.6 g) was subjected to silica gel CC eluted with a step gradient CHCl_3_–MeOH (50:1 to 0:1) system. After being analyzed by TLC, fractions A–E were obtained. Fraction A (3.0 g) was fractionated by silica gel CC eluted with step gradient *n*-hexane-aectone (8:1 to 1:1) eluents to obtain alkaloid-containing fractions A1 and A2. Compound **10** (71 mg) was precipitated from A1 in MeOH. The mother liquor (335 mg) was separated by Sephadex LH-20 (MeOH) to afford compounds **10** (27 mg) and **14** (80 mg), and a mixture, which was further purified by semipreparative HPLC eluted with 35% MeOH–H_2_O containing 0.1% trifluoroacetic acid (TFA) to give compound **11** (13 mg, *t*_R_ 17.5 min). The purification of fraction A2 (650 mg) through Sephadex LH-20 (acetone) provided two subfractions, A21–A22. A21 (150 mg) was isolated on silica gel CC eluted with CHCl_3_–MeOH (10:1) to yield compound **6** (15 mg). Compounds **7** (9 mg, *t*_R_ 25.2 min) and **15** (6 mg, *t*_R_ 16.5 min) were purified from A22 by semipreparative HPLC with 20% MeCN–H_2_O (+0.1% TFA) as an eluent. Compound **8** (1.8 g) was precipitated from fraction B. The left mother liquor (13.5 g) was performed CC on ODS eluted with a step gradient MeOH–H_2_O (10% to 50%) to obtain two alkaloid-containing parts B1 and B2. Compounds **8** (320 mg) and **9** (50 mg) were precipitated from B1 and B2, respectively. Fractions C (3.1 g), D (3.9 g), and E (9.8 g) were isolated by ODS CC with a step gradient MeOH–H_2_O (10% to 60%) eluent, respectively. Compound **1** (103 mg) was precipitated from the 30% MeOH fraction of C in MeOH. The following 40% MeOH fraction of D was further purified by semipreparative HPLC using 25% MeCN–H_2_O (+0.1% TFA) as eluent to give compound **12** (32 mg, *t*_R_ 22.6 min). Compound **4** (300 mg) was crystalized from the 30% MeOH fraction of D in MeOH. Subfractions E1 (830 mg) and E2 (310 mg) from fraction E were first separated by Sephadex LH-20 (MeOH) to afford E1A, E1B, and E2A, respectively. Compounds **17** (3 mg, 20% MeOH–H_2_O, +0.1% TFA, *t*_R_ 18.1 min), **2** (15 mg, 10% MeCN–H_2_O, +0.1% TFA, *t*_R_ 23.5 min), and **3** (4 mg, 25% MeCN–H_2_O, +0.1% TFA, *t*_R_ 20.7 min) were further purified from E1A, E1B, and E2A by semipreparative HPLC, respectively.

The *n*-BuOH (60.5 g) extraction was applied to macroporous resin D-101 CC eluted with a step gradient of EtOH-H_2_O (0 to 100%) solution. TLC analysis showed only the 20% EtOH fraction included alkaloids. Therefore, the 20% EtOH fraction (10.8 g) was submitted to NH-gel CC eluted with a gradient of CHCl_3_–MeOH (6:1 to 1:1) to give fractions F–I. Fraction F (1.2 g) was first isolated by Sephadex LH-20 (MeOH) and then purified by preparative HPLC (40% MeOH–H_2_O, +0.1% TFA) to yield compound **5** (520 mg, *t*_R_ 20.3 min). Fraction C (1.5 g) was chromatographed through an ODS column with a step gradient of MeOH–H_2_O (10% to 30%) to afford a mixture, which was further purified by preparative HPLC (15% MeCN–H_2_O, +0.1% TFA) to give compounds **13** (371 mg, *t*_R_ 18.3 min) and 1**6** (152 mg, *t*_R_ 11.6 min), respectively. NMR spectra of compounds **1**−**3** can be found in the Supplementary Materials.

11-Hydroxyburnamine (**1**): yellowish, amorphous powder; ${\left[\alpha \right]}_{D}^{20}$ − 117.5 (MeOH, *c* 0.04); UV (MeOH) *λ* max (log *ɛ*) 207 (4.75), 293 (3.68) nm; ^1^H and ^13^C NMR data, Table 1; HRESIMS *m*/*z* 385.1766 [M + H]^+^ (calcd for C_21_H_25_N_2_O_5_, 385.1758). 

Rauvoyunnanine A (**2**): yellowish, amorphous powder; ${\left[\alpha \right]}_{D}^{20}$ + 74 (MeOH, *c* 0.1); UV (MeOH) *λ* max (log *ɛ*) 203 (4.44), 275 (3.85) nm; ^1^H and ^13^C NMR data, Table 1; HRESIMS *m*/*z* 327.1676 [M + H]^+^ (calcd for C_19_H_23_N_2_O_3_, 327.1703).

Rauvoyunnanine B (**3**): yellowish, amorphous powder; ${\left[\alpha \right]}_{D}^{20}$ + 151 (MeOH, *c* 0.1); UV (MeOH) *λ* max (log *ɛ*) 252 (4.38), 307 (4.07), 367 (3.44) nm; ^1^H and ^13^C NMR data, Table 1; HRESIMS *m*/*z* 437.1274 [M + Na]^+^ (calcd for C_22_H_23_N_2_O_4_ClNa, 437.1239).

### 3.4. Assay for Inhibitory Activity on T Cell Proliferation *[17]*

#### 3.4.1. T Cell Preparation 

Peripheral blood mononuclear cells (PBMCs) were isolated from three healthy donors by density-gradient centrifugation using Lymphoprep. The cells were cultured in Roswell Park Memorial Institute (RPMI) 1640 supplemented with 10% FBS. The T cells were isolated using the Pan T Cell Isolation Kit II Human with negative selection. Then, the T cells were stained using the PE-anti-CD3 antibody (BD PharMingen, San Diego, CA, USA), and the number of stained cells was determined using flow cytometry (Acurri C6, Becton Dickinson, San Jose, CA, USA). The T cells were at 95% purity during the following experiments.

#### 3.4.2. Cell Proliferation Assay

Flow cytometry was used to probe T cell proliferation using 5-carboxyfluorescein diacetate succinimide ester (CFSE, Molecular Probes, Eugene, OR, USA)-labeling. Briefly, human naïve T cells or PBMCs (10^6^ cells/mL) were stained with 2.5 μM CFSE at 37 °C for 10 min, washed with PBS twice and re-suspended in RPMI 1640 medium containing 10% FBS. Then, the labeled naïve T cells (10^6^ cells/mL) were activated by plate-bound anti-CD3 (2 μg/mL, HIT3a clone) and soluble anti-CD28 (1 μg/mL, CD28.2 clone, BD PharMingen). The labeled PBMCs (10^6^ cells/mL) were activated with equal numbers of PBMCs irradiated with 3000 rad from another person. Subsequently, the proliferation of the T cells activated by the anti-CD3/anti-CD28 antibodies or alloantigen was measured by flow cytometry after stimulation for 72 h incubated with or without different concentrations of isolated compounds. The cells without a stimulation or drug served as the negative control, and the positive control was the cells with the stimulation but without the drug.

#### 3.4.3. Statistical Analysis 

The inhibitory concentrations of the compounds that reduced cell proliferation by 50% (IC_50_) values were calculated using GraphPad Prism 6 (GraphPad, San Diego, CA, USA). One-way analysis of variance (ANOVA) with Dunnett comparisons on post-tests were used to analyze data and compare groups. The results are expressed as the mean ± S.E.M. *P* < 0.05 was considered to be statistically significant.

## 4. Conclusions

In this study, a new picraline-type alkaloid (**1)**, a new sarpagine-type alkaloid (**2**), and a new serpentine-type alkaloid (**3**) were obtained from the whole plants of *R. yunnanensis*. Their structures were extensively elucidated by HRESIMS, 1D and 2D NMR, and UV analysis. Compounds **1** and **6** showed moderate immunosuppressive activity on T cell proliferation. Previous bioactivity studies of reserpine (**6**) mainly focused on antihypertension [14]. Although reserpine induced suppression on delayed hypersensitivity and contact sensitivity [15], its immunosuppressive activity on T cell proliferation was reported for the first time. The discovery of these new compounds enriched the chemical diversity of monoterpene indole alkaloids and also provided a potential immunosuppressant for further mechanism study. 

## Figures and Tables

**Table 1:** ^1^H and ^13^C NMR spectroscopic data of compounds **1**–**3**. **1** in C_5_H_5_N-*d*_5_, **2** and **3** in MeOH-*d*_4_.

Position	1		2		3	
	*δ*_C_	*δ*_H_ (*J* in Hz)	*δ*_C_	*δ*_H_ (*J* in Hz)	*δ*_C_	*δ*_H_ (*J* in Hz)
2	108.7		132.7		135.7	
3	53.0	3.89, m	66.8	5.02, d (9.8)	133.1	
5	87.8	4.92, d (1.6)	70.7	3.74, m	133.2	8.24, s
6	46.5	3.44, d (13.6) 2.62, dd (13.6, 2.1)	25.0	3.37, brd (12.2) 2.95, d (16.1)	139.5	
7	53.6		102.0		130.8	
8	125.6		128.5		121.0	
9	128.0	7.61, d (8.1)	103.6	6.87, br s	125.4	8.36, d (8.2)
10	108.1	6.71, dd (8.1, 1.8)	152.3		123.5	7.50, m
11	159.8		113.5	6.74, d (7.7)	132.4	7.79, overlap
12	100.1	6.79, d (1.8)	113.2	7.21, d (8.5)	114.1	7.79, overlap
13	153.1		133.5		145.3	
14	22.9	2.28, d (14.0) 1.97, m	34.7	2.59, m 2.17, br d (9.7)	31.6	4.68, m 3.14, m
15	33.6	3.85, m	27.5	3.05, br s	26.0	3.07, m
16	59.1		46.1	2.27, m	107.2	
17	64.6	4.22, d (11.6) 4.01, d (11.6)	63.8	3.55, d (7.1)	156.3	7.72, d (1.2)
18	13.8	1.64, d (6.5)	13.2	1.70, d (6.5)	14.2	1.36 d (6.7)
19	119.7	5.28, m	122.9	5.62, m	72.9	4.77, m
20	140.1		129.8		38.5	2.70, m
21	47.5	3.90, d (17.8) 3.17, d (17.8)	69.8	4.66 d (14.0) 4.36 d (14.0)	57.4	4.87, m 4.61, t (13.0)
22	175.6				168.5	
COOCH_3_	51.7	3.70, s			51.9	3.82, s
OCH_3_					64.3	3.60, s

**Table 2:** IC_50_ values of compounds **1**–**17** (μM) and total alkaloids (µg/mL) from *R. yunnanensis* against T cell proliferation.

Sample	IC_50_
**1** **2–5**	5.9 ± 0.8 >50
**6** **7–17**	5.0 ± 0.5 >50
**Total alkaloids**	18.5 ± 0.8

## Supplementary Materials

Supplementary data (NMR spectra of compounds **1**−**3**) to this article can be found online.
