# Supplementary material for: Bioactivity-Guided Isolation and Identification of New and Immunosuppressive Monoterpenoid Indole Alkaloids from Rauvolfia yunnanensis Tsiang
Source: Molecules. 2019 Dec 13;24(24):4574. doi: 10.3390/molecules24244574 (PMC6943595; doi:10.3390/molecules24244574)

Supplementary data

Bioactivity-guided Isolation and Identification of New and Immunosuppressive Monoterpenoid Indole Alkaloids from *Rauvolfia yunnanensis* Tsiang

Li-Mei Li[1,2,*], Shun-Dong Shi[2], Yang Liu[2], Qiang Zou[2]

[1] College of Pharmacy, Southwest University for Nationalities, Chengdu 610041, Sichuan, China

[2] Research Center, Chengdu Medical College, Chengdu 610500, Sichuan, China; shisd@kelun.com (S.-D. S.); scunn519@gmail.com (Y. L.); qiangzou99@gmail.com (Q. Z.)

[*] Correspondence: 21800008@swun.edu.cn (L.-M. L.).

Content

Figure S1. ^{1}H NMR spectrum of compound **1** (600 MHz, C_5H_5N-d_5).

Figure S2. ^{13}C NMR spectrum of compound **1** (150 MHz, C_5H_5N-d_5).

Figure S3. HSQC spectrum of compound **1** (600 MHz, C_5H_5N-d_5).

Figure S4. HMBC spectrum of compound **1** (600 MHz, C_5H_5N-d_5).

Figure S5. NOESY spectrum of compound **1** (600 MHz, C_5H_5N-d_5).

Figure S6. ^{1}H NMR spectrum of compound **2** (600 MHz, MeOH-d_4).

Figure S7. ^{13}C NMR spectrum of compound **2** (150 MHz, MeOH-d_4).

Figure S8. HSQC spectrum of compound **2** (600 MHz, MeOH-d_4).

Figure S9. HMBC spectrum of compound **2** (600 MHz, MeOH-d_4).

Figure S10. NOESY spectrum of compound **2** (600 MHz, MeOH-d_4).

Figure S11. ^{1}H NMR spectrum of compound **3** (600 MHz, MeOH-d_4).

Figure S12. ^{13}C NMR spectrum of compound **3** (150 MHz, MeOH-d_4).

Figure S13. HSQC spectrum of compound **3** (600 MHz, MeOH-d_4).

Figure S14. HMBC spectrum of compound **3** (600 MHz, MeOH-d_4).

Figure S15. NOESY spectrum of compound **3** (600 MHz, MeOH-d_4).

Figure S1. ^{1}H NMR spectrum of compound **1** (600 MHz, C_5H_5N-d_5).

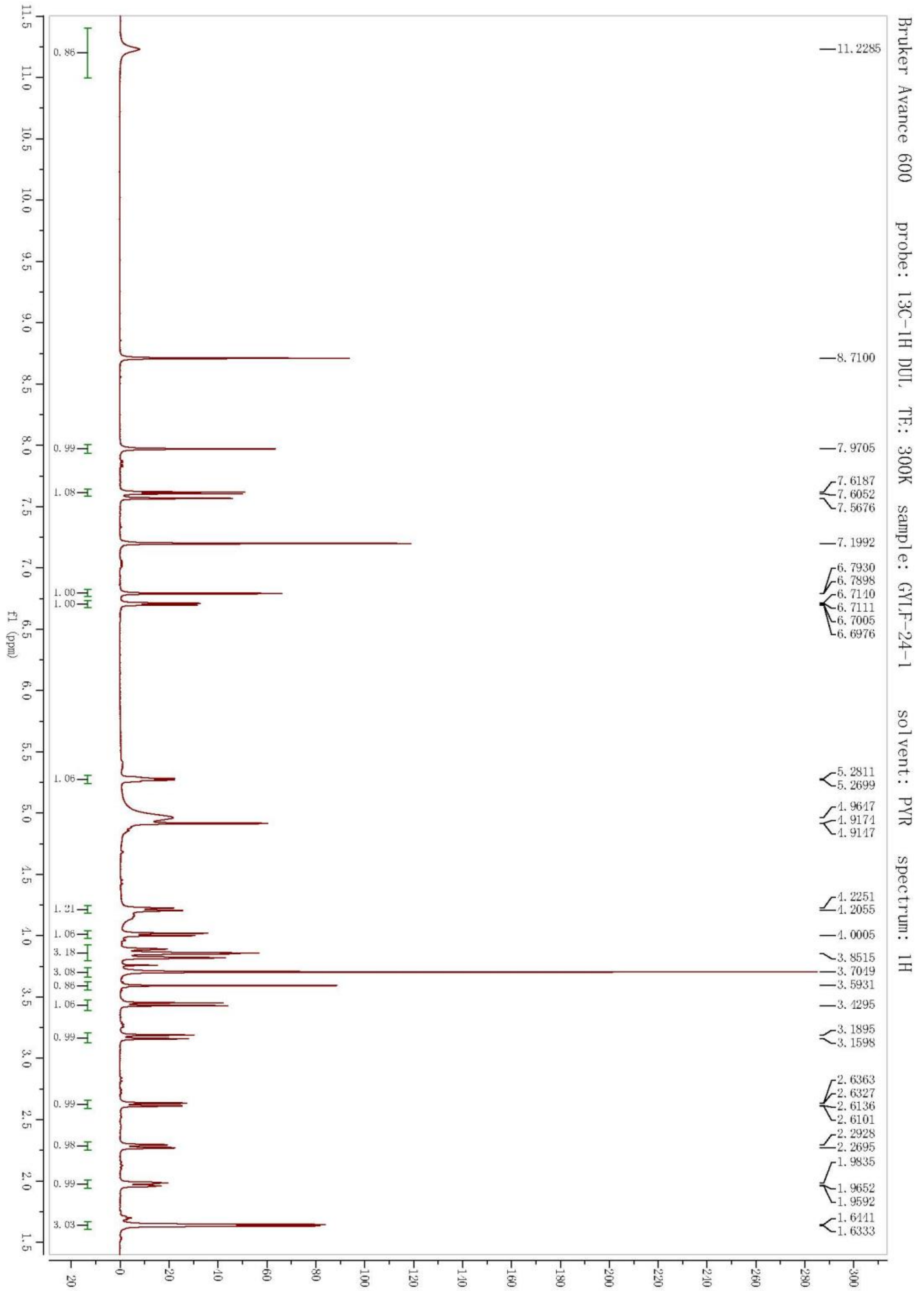

Figure S2. ^{13}C NMR spectrum of compound **1** (150 MHz, C_5H_5N-d_5).

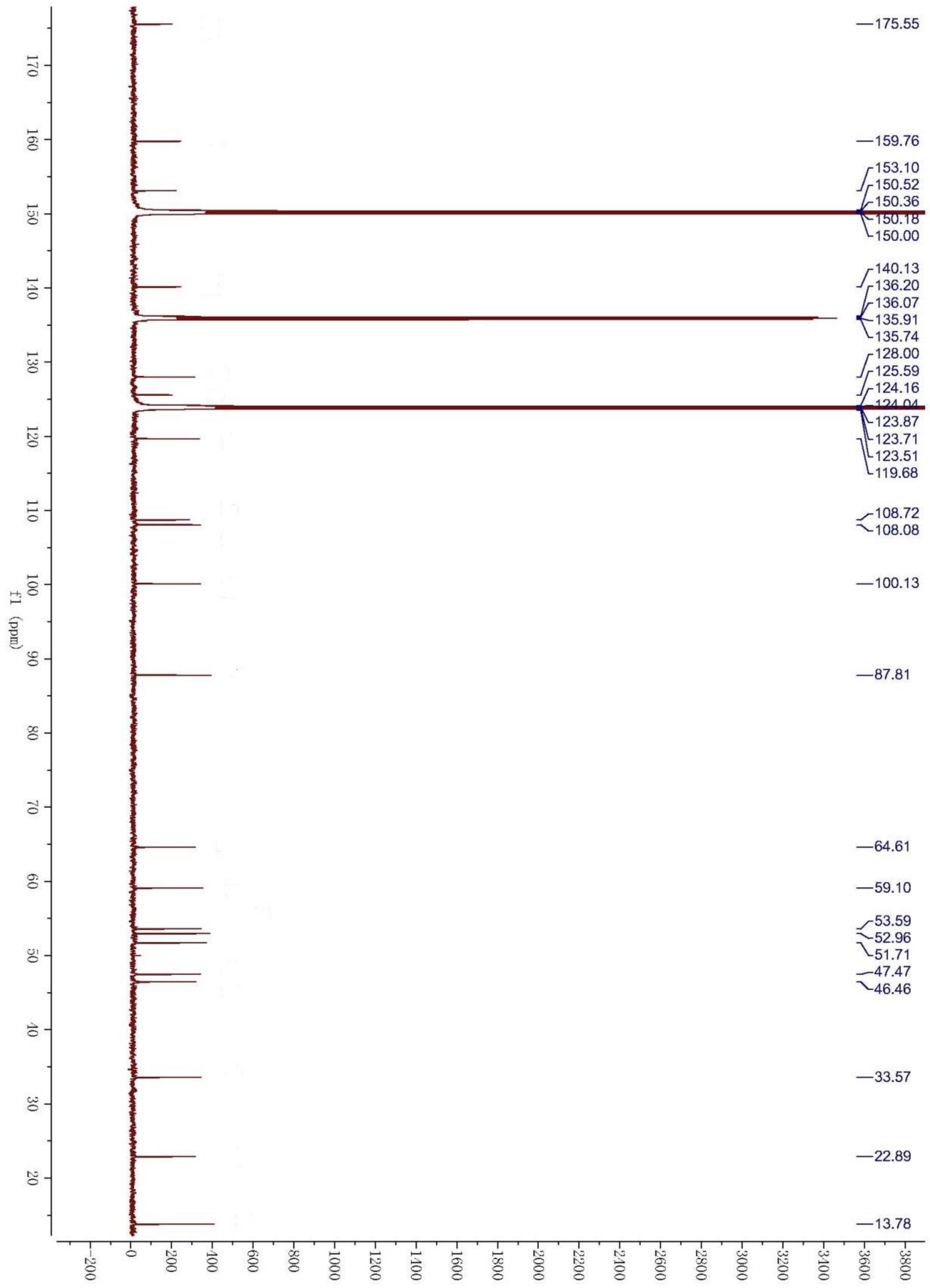

Figure S3. HSQC spectrum of compound **1** (600 MHz, C_5H_5N-d_5).

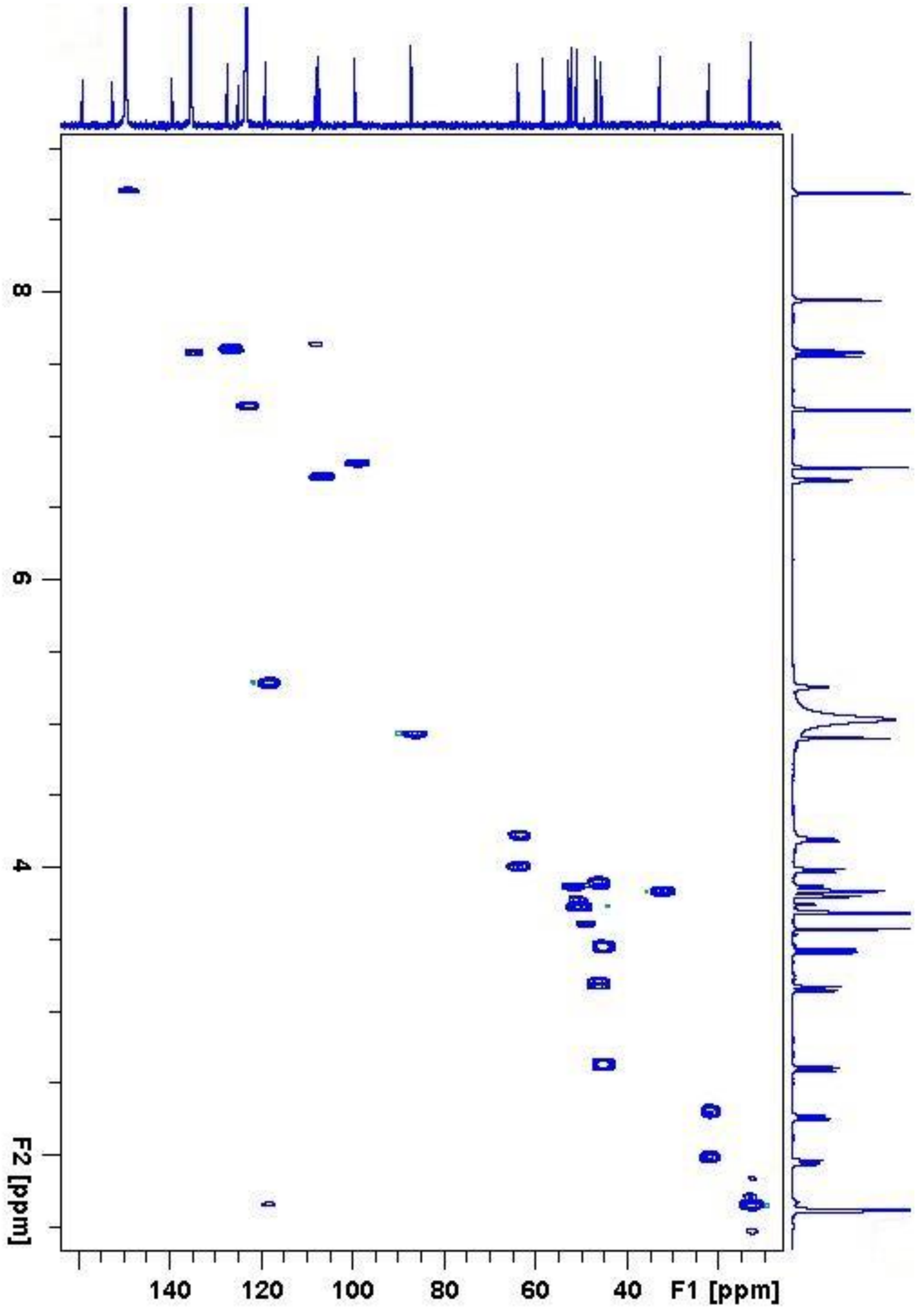

Figure S4. HMBC spectrum of compound **1** (600 MHz, C_5H_5N-*d_5*).

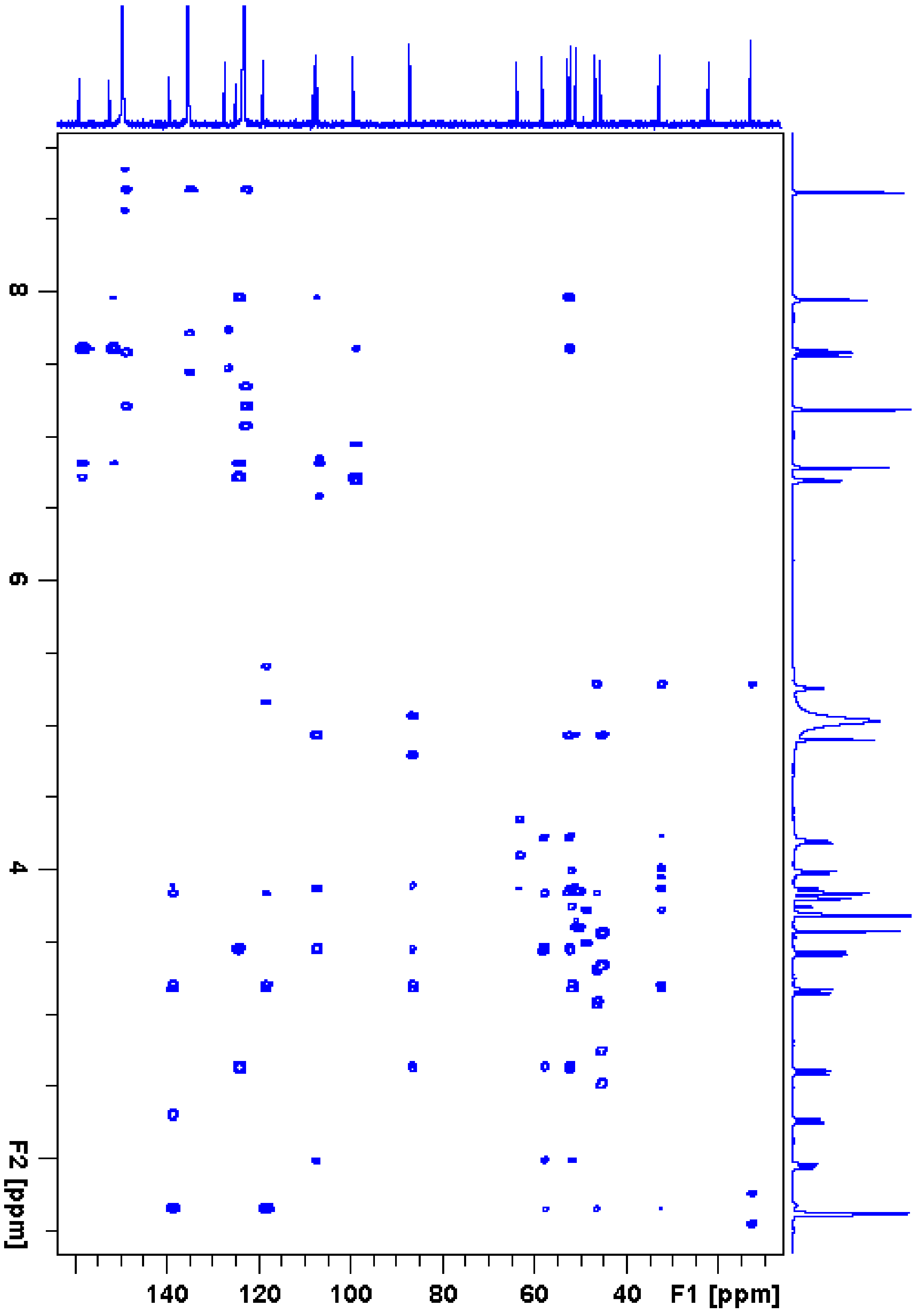

Figure S5. NOESY spectrum of compound **1** (600 MHz, C_5H_5N-d_5).

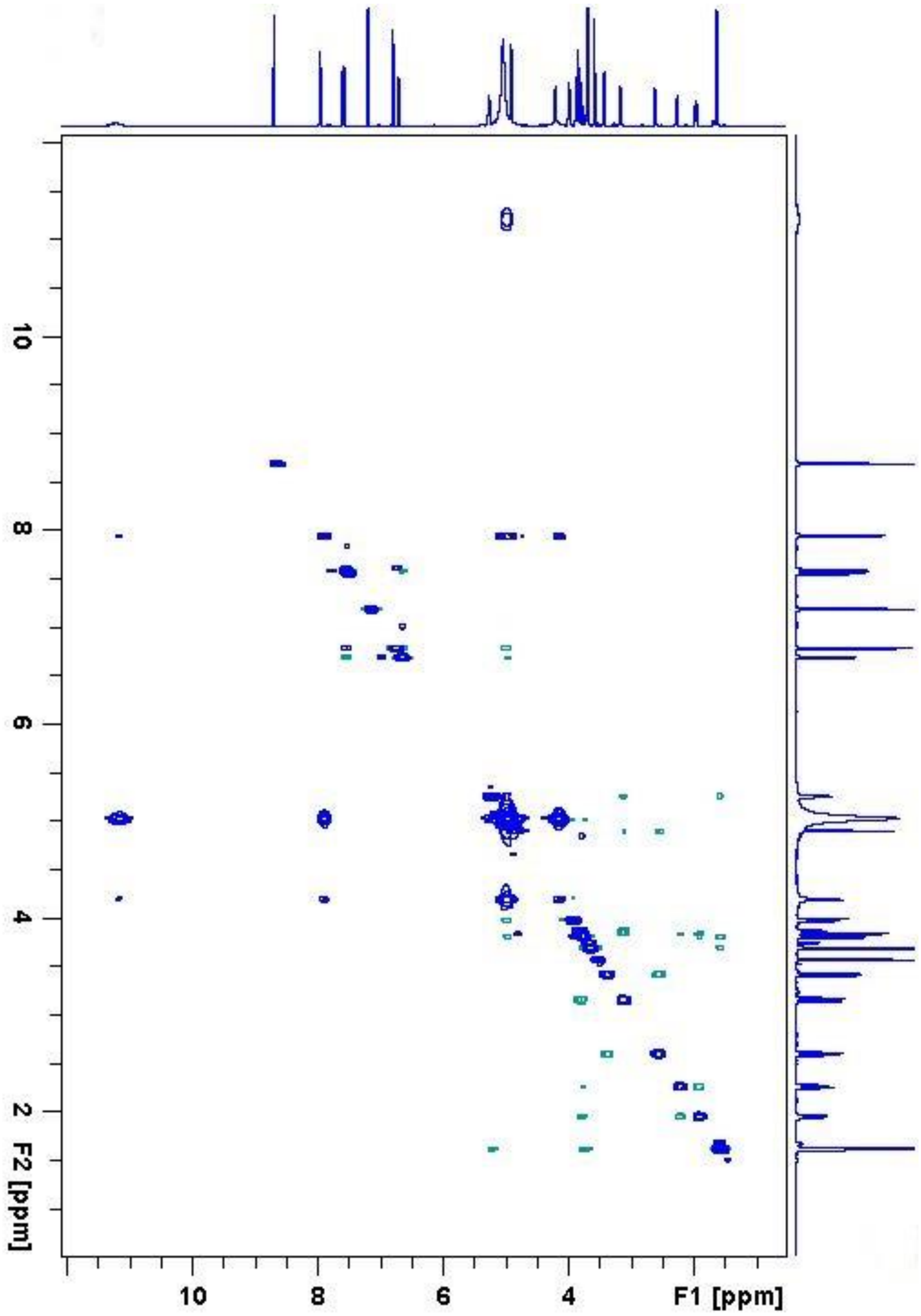

Figure S6. ^{1}H NMR spectrum of compound **2** (600 MHz, MeOH-d_4).

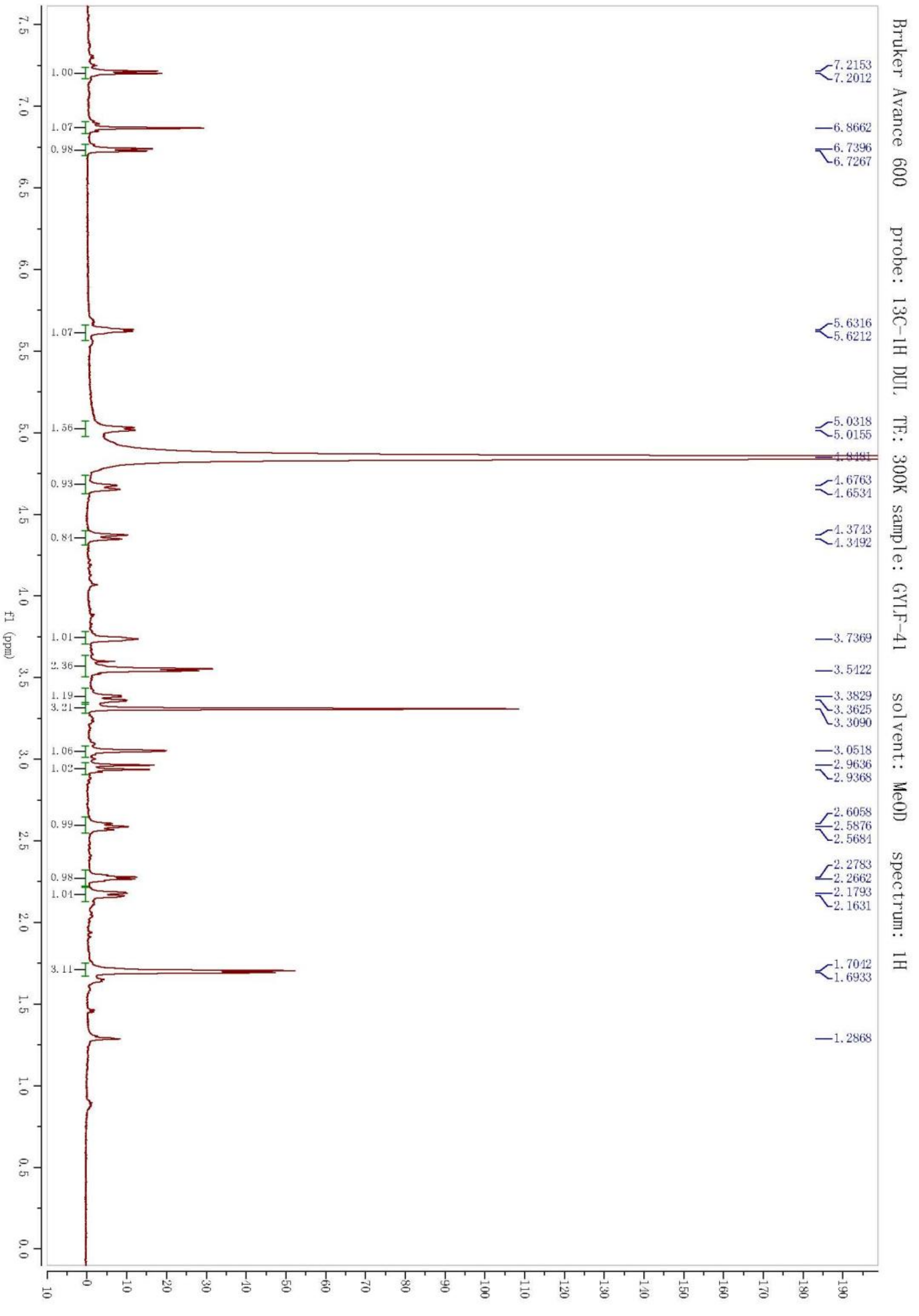

Figure S7. ^{13}C NMR spectrum of compound **2** (150 MHz, MeOH-d_4).

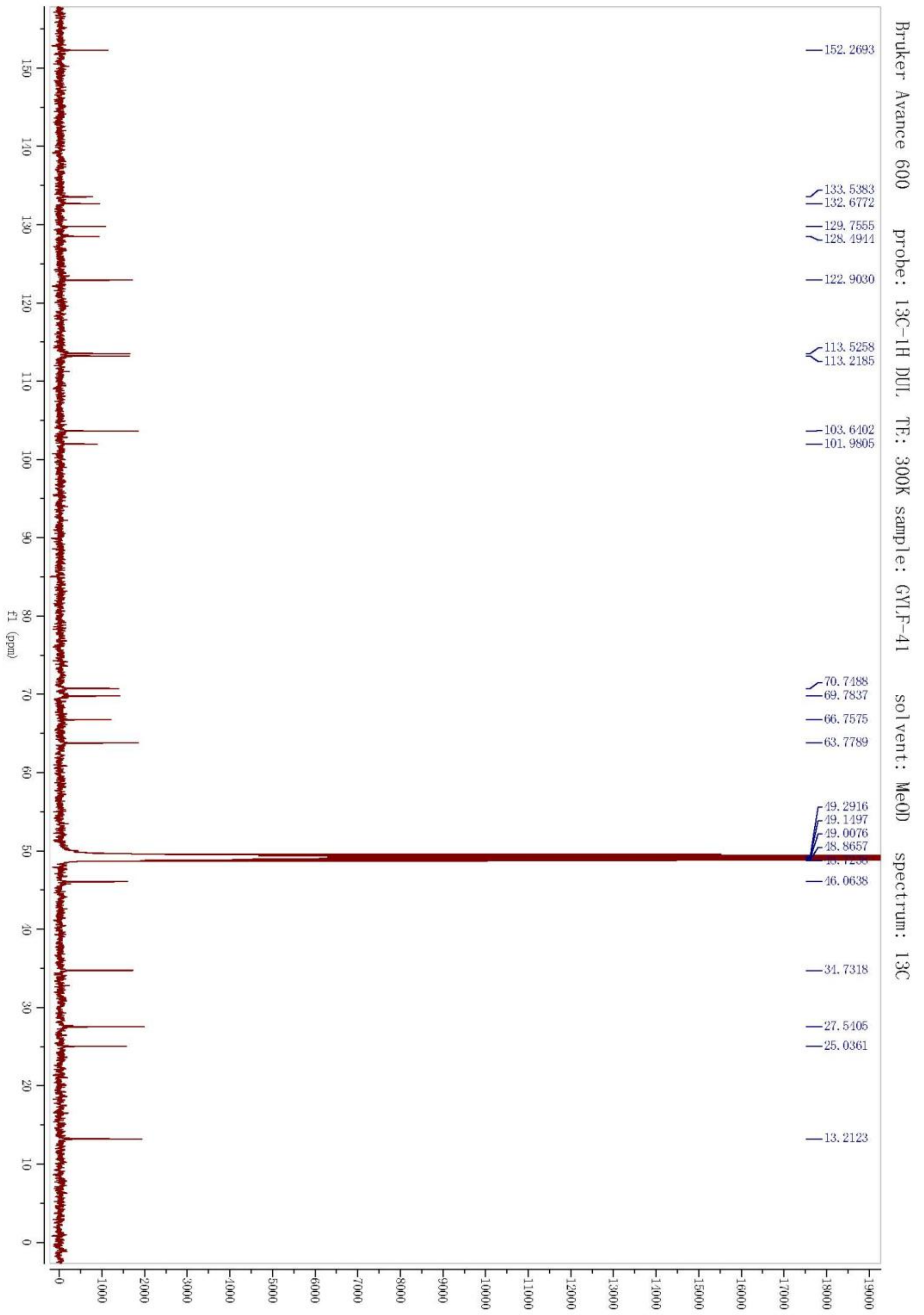

Figure S8. HSQC spectrum of compound **2** (600 MHz, MeOH-d_4).

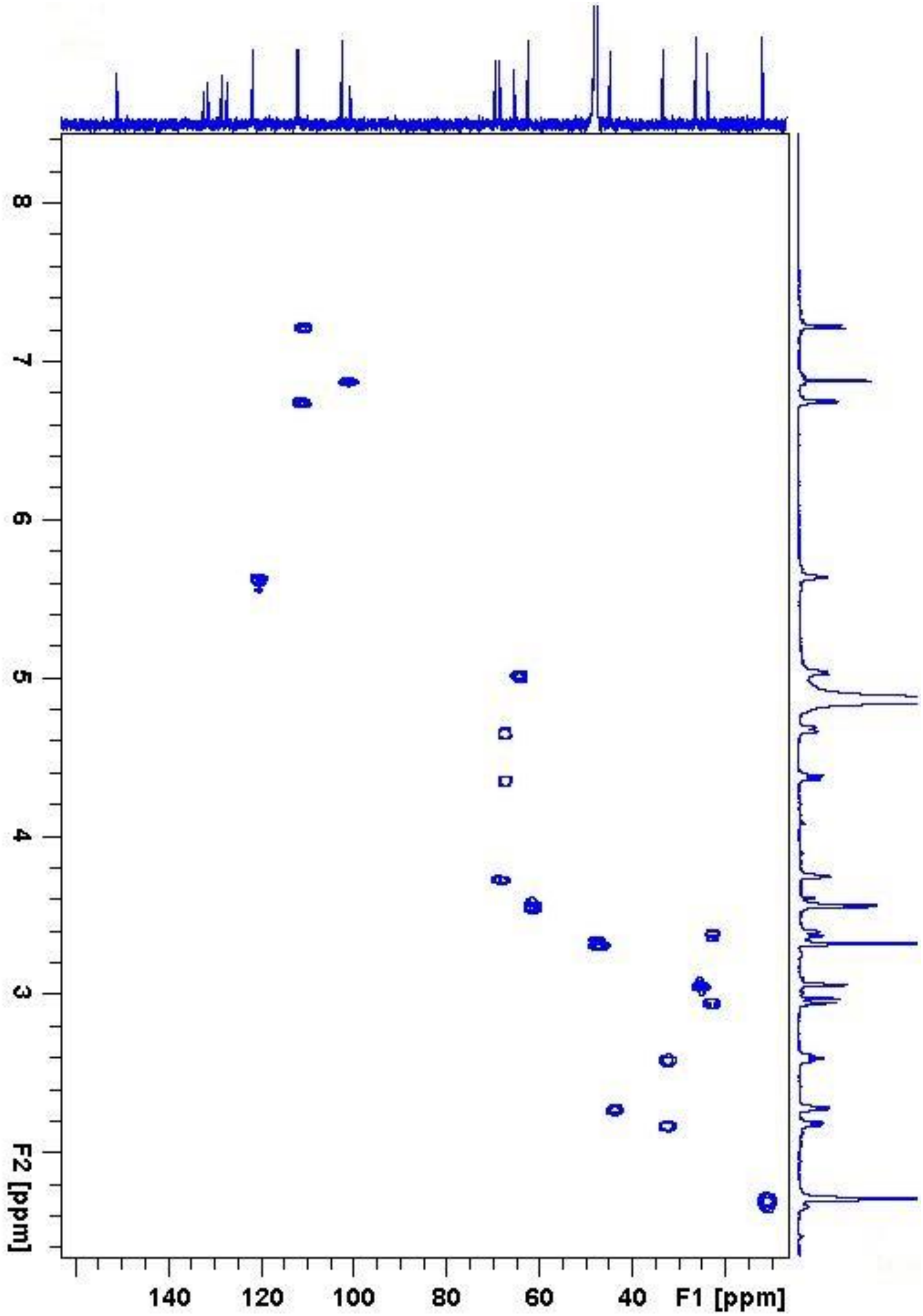

Figure S9. HMBC spectrum of compound **2** (600 MHz, MeOH-d_4).

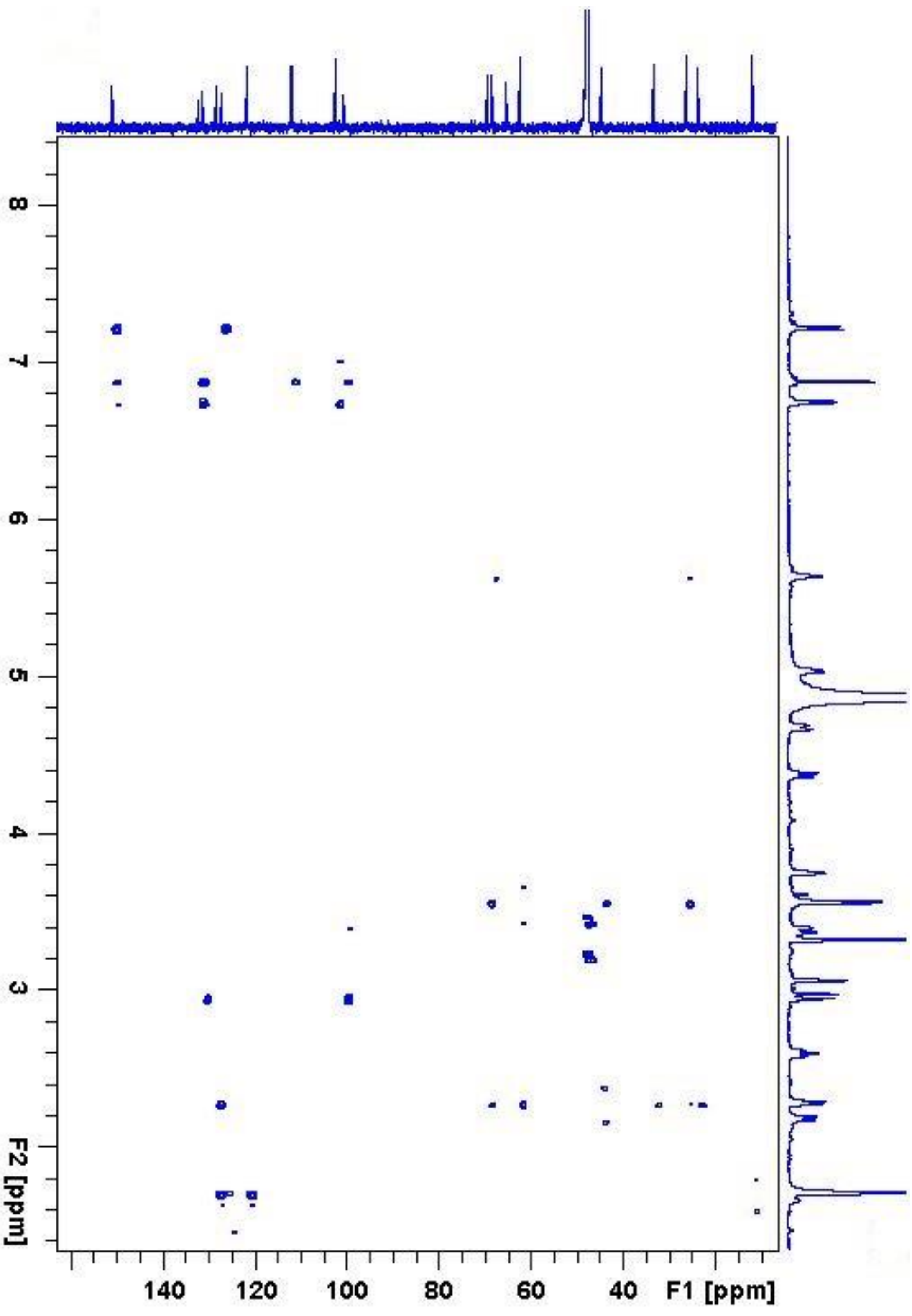

Figure S10. NOESY spectrum of compound **2** (600 MHz, MeOH-d_4).

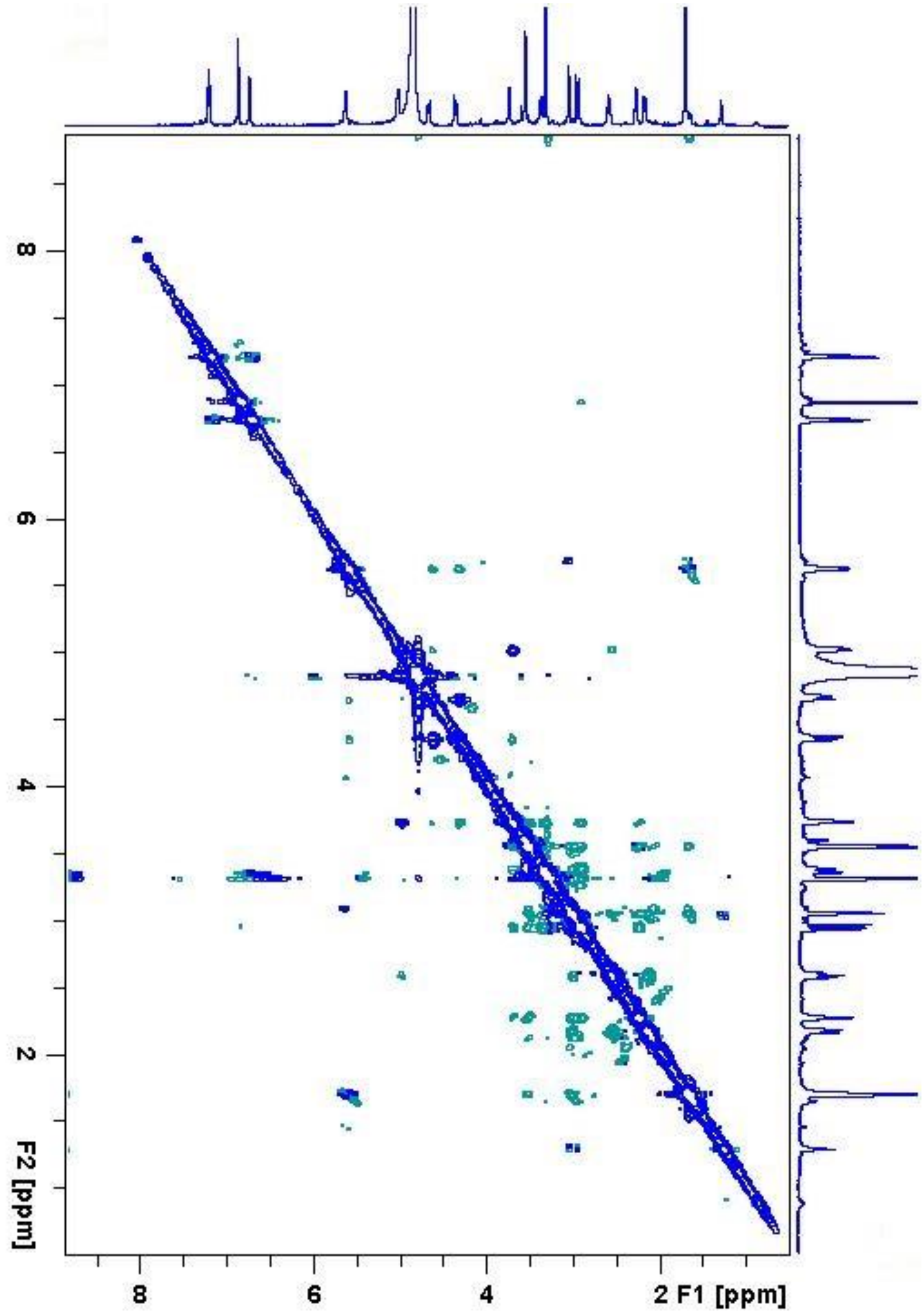

Figure S11. ^{1}H NMR spectrum of compound **3** (600 MHz, MeOH-d_4).

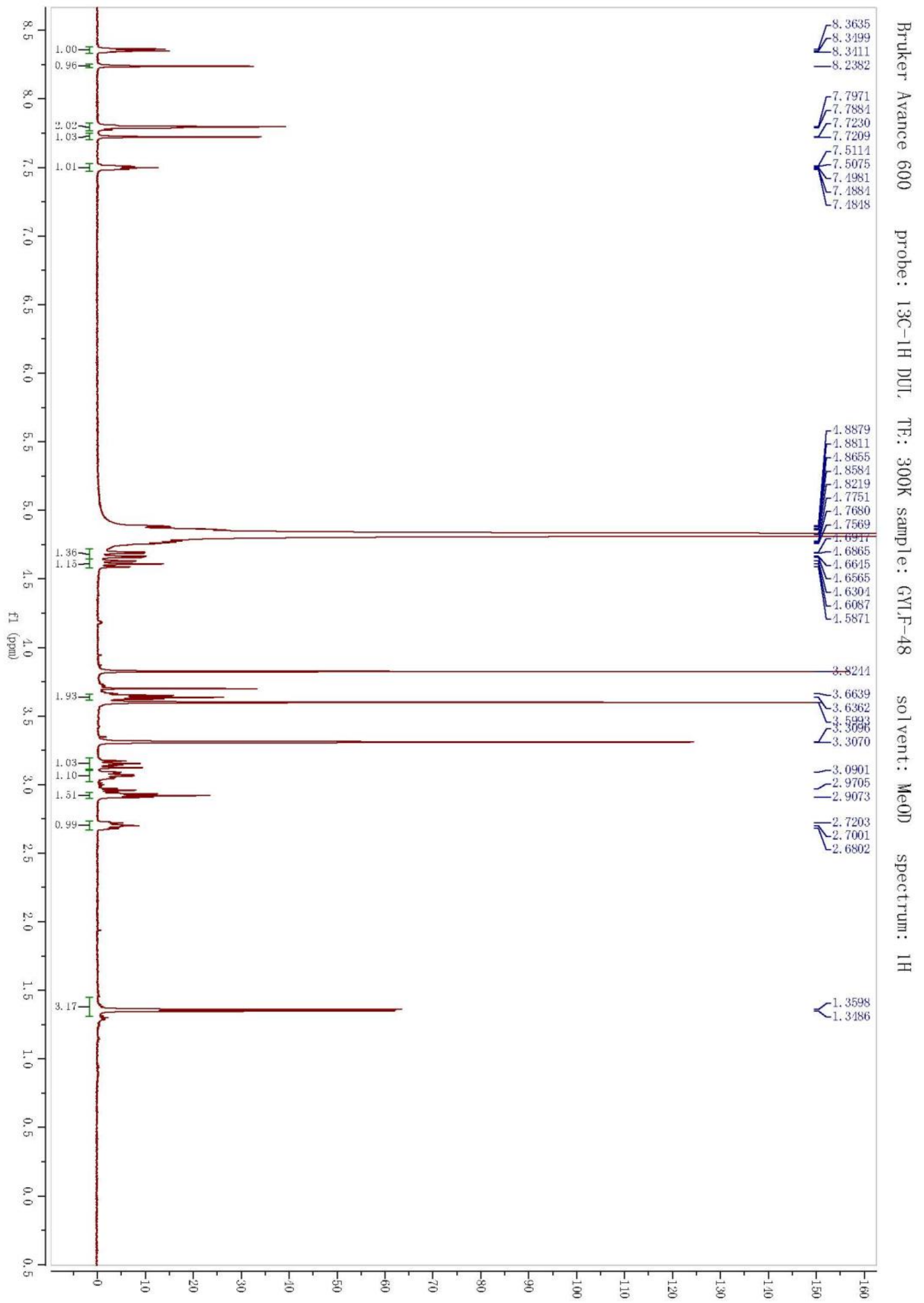

Figure S12. ^{13}C NMR spectrum of compound **3** (150 MHz, MeOH-d_4).

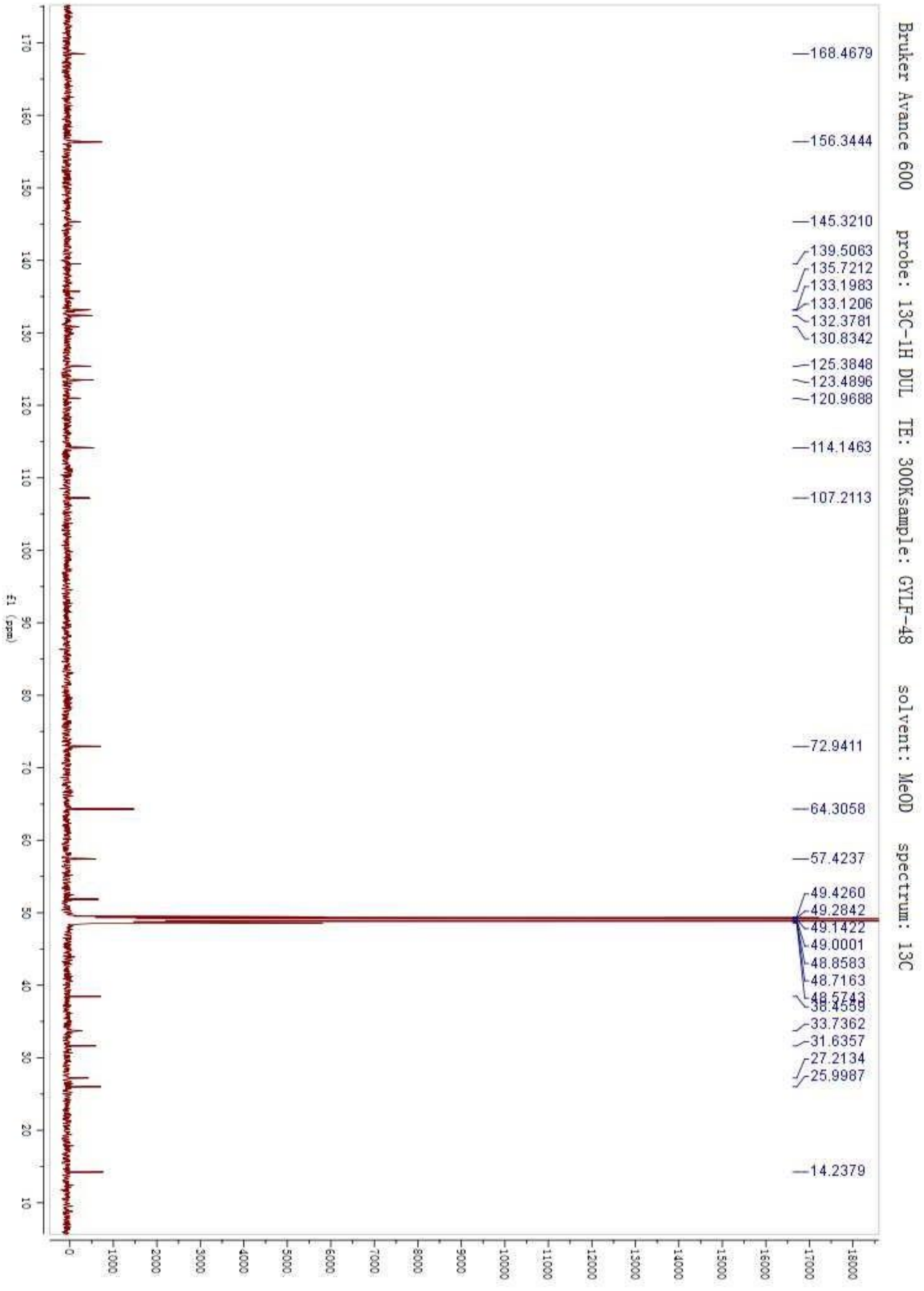

Figure S13. HSQC spectrum of compound **3** (600 MHz, MeOH-d_4).

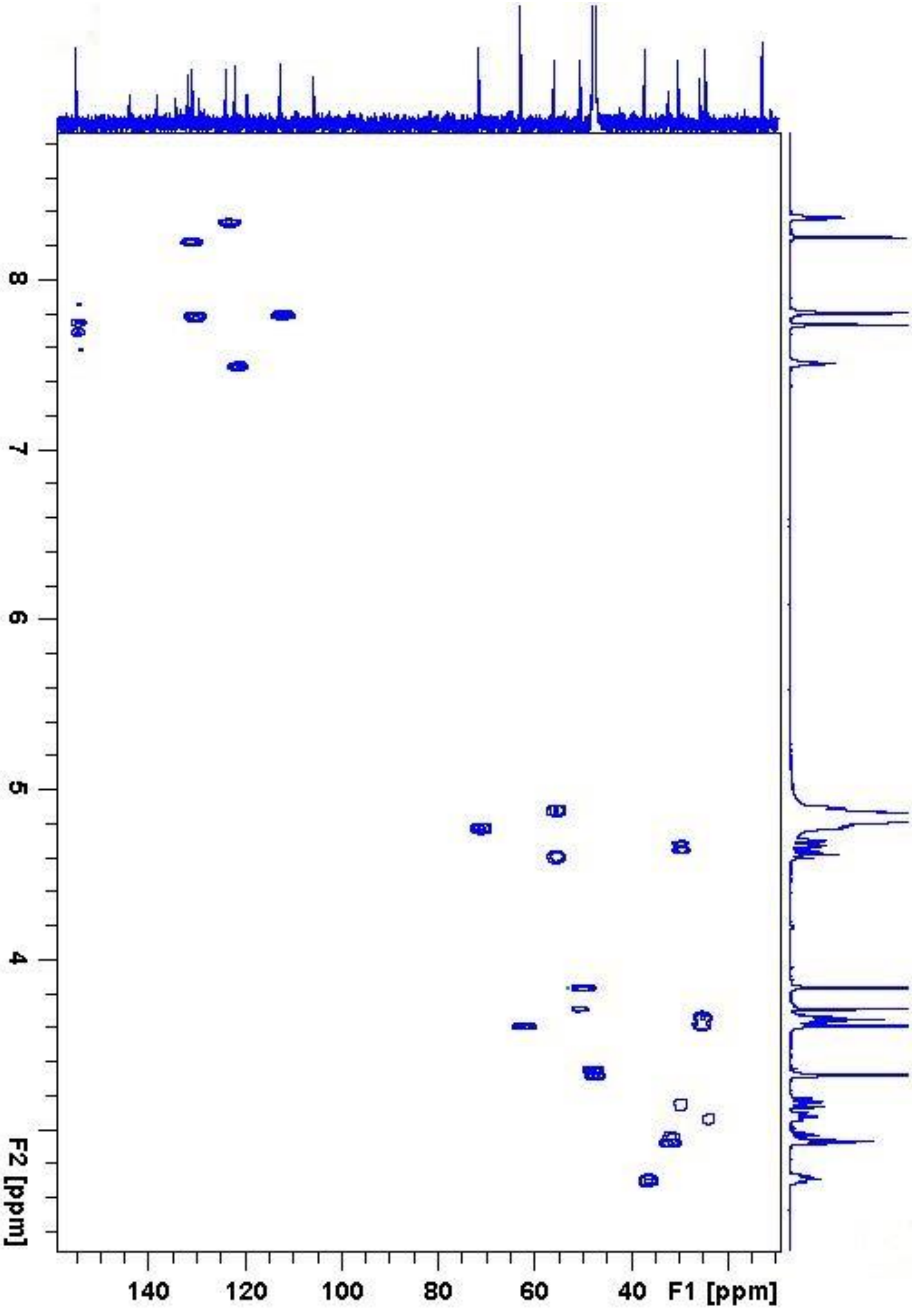

Figure S14. HMBC spectrum of compound **3** (600 MHz, MeOH-d_4).

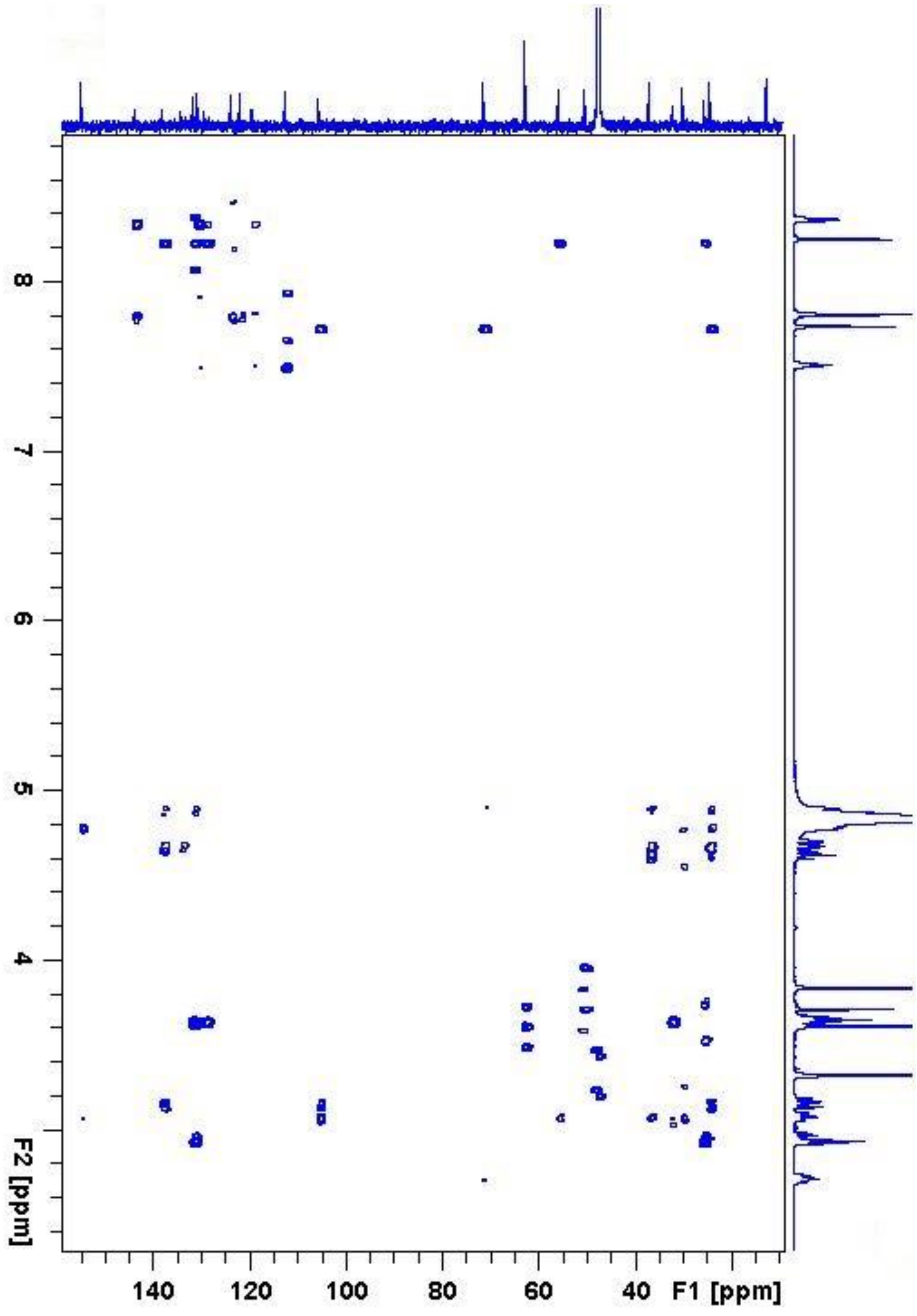

Figure S15. NOESY spectrum of compound **3** (600 MHz, MeOH-d_4).

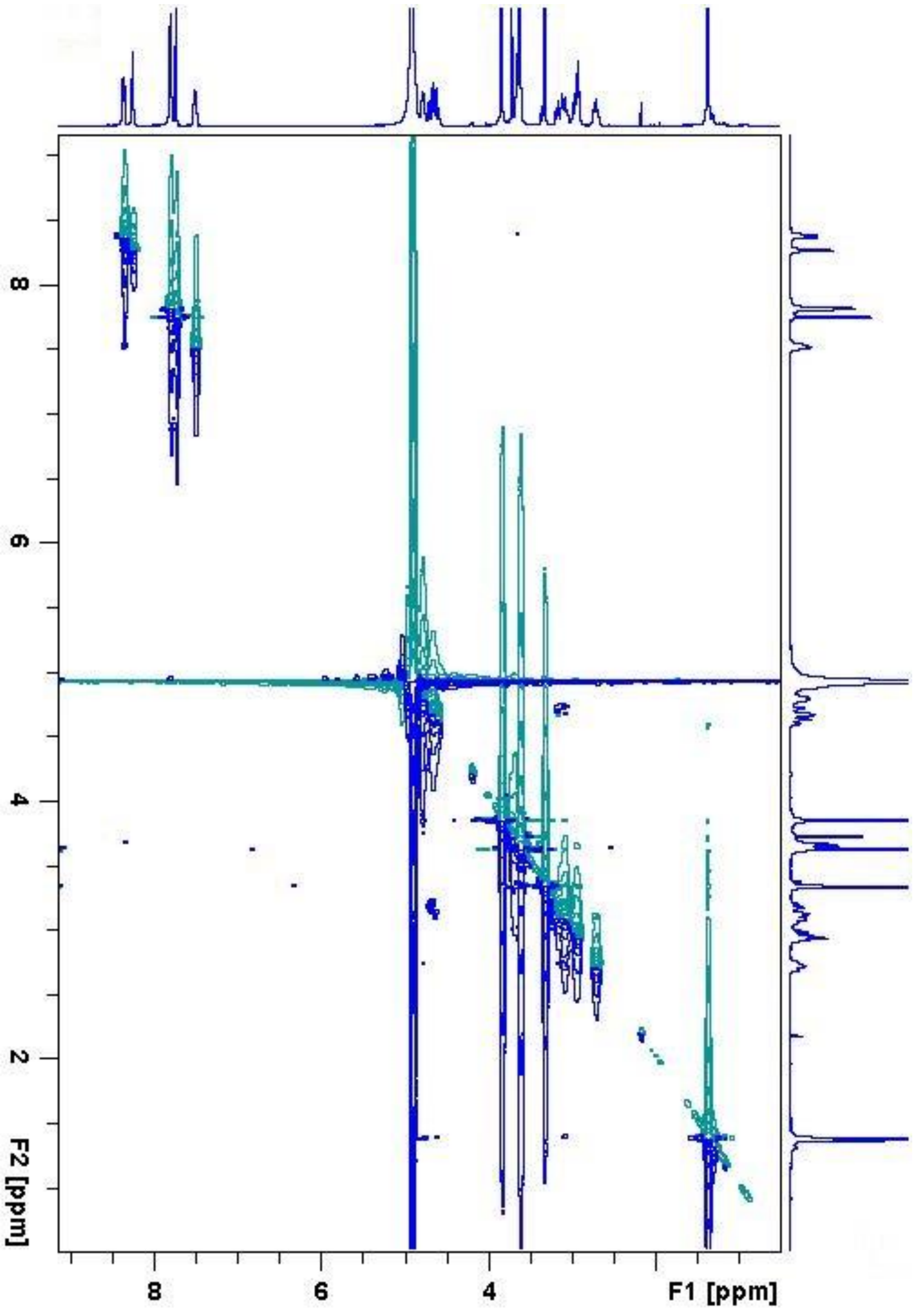

Supplement: Supplementary file 1 [file molecules-24-04574-s001.pdf]
